# Dataset of the frequency patterns of publications annotated to human protein-coding genes, their protein products and genetic relevance

**DOI:** 10.1016/j.dib.2019.104284

**Published:** 2019-07-18

**Authors:** Matthias Zwick, Oliver Kraemer, Adrian J. Carter

**Affiliations:** a Computational Biology, Boehringer Ingelheim, 88400 Biberach an der Riß, Germany; b Discovery Research Coordination, Boehringer Ingelheim, 55216 Ingelheim Am Rhein, Germany

**Keywords:** Human proteome, Human genome, Scientific publications, Data integration, Genome-Wide Association Studies (GWAS), Online Mendelian Inheritance in Man (OMIM)

## Abstract

We present data concerning the distribution of scientific publications for human protein-coding genes together with their protein products and genetic relevance. We annotated the gene2pubmed dataset Maglott et al., 2007 provided by the NCBI (National Center for Biotechnology Information) with publication years, genetic metadata corresponding to Online Mendelian Inheritance in Man (OMIM) Hamosh et al., 2005 entries and the frequency of their appearance in Genome-Wide Association Studies (GWAS) Buniello et al., 2019 provided by the European Bioinformatics Institute (EBI) using the KNIME^®^ Analytics Platform Berthold et al., 2008. The results of this data integration process comprise two datasets: 1) A dataset containing information on all human protein-coding genes that can be used to analyse the number of scientific publications in context of the potential disease relevance of the individual genes. 2) A table with the annual and cumulated number of PubMed entries. For further interpretation of the data presented in this article, please see the research article ‘Target 2035 - probing the human proteome’ by Carter et al. https://doi.org/10.1016/j.drudis.2019.06.020 Carter et al., 2019.

**Table:** Specifications Table

Subject	Chemical biology
Specific subject area	Data integration and mapping
Type of data	Tab separated files
How data were acquired	Download, integration and filtering of publicly available datasets in a KNIME workflow.
Data format	Filtered, Summarised
Parameters for data collection	Information about publications on human genes was identified via the gene2pubmed dataset. Relevance for disease phenotype was assessed via GWAS catalog and OMIM.
Description of data collection	Publicly available datasets from the NCBI (gene2pubmed, mim2gene_medgen, gene_info) and the EBI (GWAS catalog) were downloaded. Data from the GWAS catalog were filtered for p-value thresholds and after mapping of ENSEMBL gene identifiers and gene symbols to NCBI gene identifiers via Biomart, all datasets were integrated and summarised in KNIME.
Data source location	The data were gathered from the National Center for Biotechnology Information (*ftp://ftp.ncbi.nlm.nih.gov/*) and the European Bioinformatics Institute (https://www.ebi.ac.uk/gwas/*).*
Data accessibility	With the article
Related research article	A. J. Carter, O. Kraemer, M. Zwick, A. Mueller-Fahrnow, C. H. Arrowsmith, A. M. Edwards Target 2035 – probing the human proteome Drug Discovery Today

**Table:** 

**Value of the data** • The data will be useful to analyse the research activity (as evidenced by scientific publications) on human protein-coding genes and their protein products. The dataset also provides information on the potential disease relevance or phenotype of the individual genes. As shown by Carter et al. [5] the data indicate that researchers tend to focus on a relatively small, already well-studied fraction of the proteins coded by the human genome despite evidence that many understudied proteins are potentially important for human disease phenotypes. • The analysis of these data allows the identification of genes that are understudied despite a link to disease phenotypes or an association with specific disease traits. This could stimulate research and promote the development of pharmacological tools to interrogate the understudied proteins encoded by these genes. • Dataset entries have been tagged with a number of different ID types. This allows mapping to other datasets as a basis for generation of further insights. Moreover, we are also publishing the KNIME workflow that has been used to compile and integrate the data. This will allow researchers to reproduce an updated dataset at any future point in time. • The data also provides access to the frequency of scientific publications on an annual or cumulated basis.

## 1 Data

The main dataset that is provided as Supplementary File 1 and contains a list of:

(i) all human protein-coding genes with year of earliest and year of latest publication,
(ii) number of publications, number and type of OMIM entries annotated to the genes,
(iii) number of GWAS that refer to the gene as well as a selection of gene and protein identifiers to allow for easy integration with other analyses.

Table 1 provides a description of the columns in Supplementary File 1.

**Table 1:** Description of columns in Supplementary File 1.

Column name	Description
Gene_ID	NCBI gene identifier
Gene_Type	type_of_gene from NCBI gene info (e.g. protein-coding)
PMID_Count	Number of publications for this gene in gene2pubmed
OMIM_Type	Comma separated list of types of corresponding OMIM entries (gene or phenotype)
OMIM_IDs	Comma separated list of corresponding OMIM entries
OMIM_Count	Number of corresponding OMIM entries
GWAS_Count_All	Number of GWAS studies with an association to this gene
GWAS_Count_Filtered	Same as GWAS_Count_All after p-value filtering
Gene_Symbol	Gene Symbol from NCBI gene info
Gene_Description	Description from NCBI gene info
Year_Min	Earliest Publication Year
Year_Max	Latest Publication Year
Rank	Rank by number of publications and earliest publication year
Uniprot_Swissprot_IDs	Comma separated list of Uniprot and Swissprot IDs retrieved from Biomart
Interpro_IDs	Comma separated list of Interpro IDs retrieved from Biomart
PFAM_IDs	Comma separated list of PFAM IDs retrieved from Biomart

In addition, we have generated a table (Table 2) with overall PubMed entries per year, PubMed entries related to any genes and PubMed entries related to human genes. The gene-related data were additionally restricted to only protein-coding genes.

**Table 2:** Overall Publication counts in PubMed with or without restriction to human and/or protein-coding genes since 1980. All PubMed: Number of all publications in PubMed for a given year. All genes: Number of all publications in gene2pubmed for a given year. All other columns are subsets of this column according to their title and based on entries in gene2pubmed.

Year	All PubMed	gene2pubmed				
		All genes	All protein-coding genes	All human genes	All human protein-coding genes	All human protein- coding genes cumulated
2019	386983	2078	1963	1234	1143	596891
2018	1328241	35725	33809	20627	19138	595748
2017	1126444	58694	56255	35854	33852	576610
2016	1111578	66224	63898	39811	37903	542758
2015	1089521	69137	66808	41861	40013	504855
2014	1041878	70953	68839	42352	40668	464842
2013	994460	72064	70350	41403	40091	424174
2012	939943	69191	67852	39052	38110	384083
2011	875517	65992	64895	36591	35846	345973
2010	822812	64335	63422	36220	35594	310127
2009	784058	63871	63107	34891	34425	274533
2008	750728	62229	61574	33327	32929	240108
2007	710052	54356	53755	27550	27198	207179
2006	684468	47520	46900	22740	22416	179981
2005	655917	45476	44833	22038	21679	157565
2004	619734	41980	41368	21213	20838	135886
2003	583849	38925	38299	20324	19945	115048
2002	558469	37403	36800	20137	19778	95103
2001	543369	25486	24850	12437	12047	75325
2000	530063	22135	21426	9533	9065	63278
1999	493347	20030	19360	8104	7713	54213
1998	474129	17950	17365	7350	6988	46500
1997	455877	16351	15810	6453	6140	39512
1996	457769	15074	14589	5576	5307	33372
1995	448236	13739	13282	4890	4648	28065
1994	437452	12395	11956	4354	4117	23417
1993	425908	10326	9904	3647	3429	19300
1992	417212	9198	8870	3262	3098	15871
1991	412474	7489	7188	2681	2539	12773
1990	409831	6475	6190	2186	2057	10234
1989	401987	5160	4910	1737	1635	8177
1988	386221	4382	4138	1466	1381	6542
1987	367193	3546	3345	1160	1089	5161
1986	349045	2898	2728	916	856	4072
1985	334454	2605	2407	727	670	3216
1984	317553	2145	1983	549	496	2546
1983	308772	1836	1695	404	379	2050
1982	295103	1583	1423	311	268	1671
1981	283122	1386	1229	241	208	1403
1980	280381	1173	1049	202	180	1195

## 2 Experimental design, materials, and methods

We downloaded the gene2pubmed dataset [1], [6] and all other datasets mentioned below on 25 March 2019 to derive information on publications annotated to each gene. All of the data integration steps were carried out using a KNIME [4] workflow (KIME version 3.5.3) that is included as Supplementary File 2 with this publication.

To annotate all PubMed identifiers (PMID) with the corresponding publication year we used an internal PubMed index. Alternatively, this could also be done using the NCBI E-Utilities [7]. The resulting list was used to generate overall counts of PMIDs per year and was joined to the initial gene2pubmed dataset. The resulting dataset was filtered for human (*Homo sapiens*) genes via taxonomy identifier 9606.

We then joined the human gene2pubmed subset with *Homo sapiens* gene information [8] via NCBI gene identifiers (IDs) to annotate the list with information on types of genes and descriptive metadata for later interpretation, and used this information to filter the dataset for protein-coding genes only. The dataset was grouped by gene IDs to create a table containing gene IDs, gene symbols, gene description metadata, number of publications, year of earliest publication and year of latest publication.

To obtain information about disease relevance of the individual genes, we downloaded the mim2gene_medgen file [9] from the NCBI and the GWAS [3] catalog from the EBI [10]. The mim2gene_medgen dataset was used to link genes to OMIM [2] entries. Using reported and mapped genes, upstream, downstream and SNP gene IDs we created a list of potentially relevant genes per study, mapped to NCBI gene IDs. The GWAS catalog data were filtered for p-values < 10^−6^ to select for potentially more reliable hits. Mim2gene_medgen data were mapped directly via NCBI gene IDs, for the GWAS catalog the gene symbols and Ensembl gene identifiers were mapped to NCBI gene IDs using Biomart and Ensembl version 86 [11]. Descriptive metadata (OMIM IDs, number of GWAS studies) from those tables and additional protein identifiers (Uniprot, Swissprot, Interpro and PFAM) obtained via Biomart were also added to the dataset.

Finally, genes were ranked according to their highest number of annotated publications and earliest publication year to generate the dataset that is provided with this publication as Supplementary File 1.

We also merged the global PMID to publication year table with the complete gene2pubmed dataset via PMID and subsequently with the gene information file for all species [12] via gene IDs to obtain the overall yearly publication counts for PubMed with or without restriction to human and/or protein-coding genes (Table 2).
